# The Toxicity and Detoxifying Mechanism of Cycloxaprid and Buprofezin in Controlling *Sogatella furcifera* (Homoptera: Delphacidae)

**DOI:** 10.1093/jisesa/iev077

**Published:** 2015-07-14

**Authors:** Xiaoli Chang, Yongda Yuan, Tianshu Zhang, Dongsheng Wang, Xingbin Du, Xiangwen Wu, Haixia Chen, Yaozhong Chen, Yuetong Jiao, Haiyuan Teng

**Affiliations:** ^1^Institute of Ecological and Environmental Protection, Shanghai Academy of Agricultural Sciences, Shanghai, 201403, China; ^3^Department of Plant Protection, Shanghai Agricultural Technology Extension and Service Center, Shanghai, 201103, China

**Keywords:** cycloxaprid, buprofezin, *Sogatella furcifera*, insecticidal activity, detoxifying enzyme

## Abstract

The effects of cycloxaprid (a modified neonicotinoid insecticide) and buprofezin (a thiadiazine insecticide) on mortality of the white-backed planthopper (WBPH), *Sogatella furcifera*, were determined in laboratory assays. Cycloxaprid killed WBPH nymphs and adults but buprofezin killed only nymphs, and cycloxaprid acted faster than buprofezin. One day after infestation, mortality of third-instar nymphs was >65% with cycloxaprid at 125 mg liter^−1^ but was <38% with buprofezin at 148 mg liter^−1^. By the 4th day after infestation, however, control of nymphs by the two insecticides was similar, and cycloxaprid at 125 mg liter^−1^ caused ≥80% mortality of adults but buprofezin at 148 mg liter^−1^ (the highest rate tested) caused almost no adult mortality. LC_50_ values for cycloxaprid were lowest with nymphs, intermediate with adult males, and highest with adult females. Although buprofezin was slower acting than cycloxaprid, its LC_50_ for nymphs 5 d after infestation was 3.79-fold lower than that of cycloxaprid. Mean carboxylesterase (CarE) specific activity of nymphal WBPH treated with cycloxaprid and buprofezin was higher than that of control, but there was no significant difference between cycloxaprid and control (no insecticide), and it was significantly higher for buprofezin than those of cycloxaprid and control. For glutathione S-transferase and mixed function oxygenase, the specific activity of nymphal WBPH treated with buprofezin was significantly higher than those of cycloxaprid and control, too.

Cycloxaprid is an oxabridged cis-configuration neonicotinoid insecticide that was first reported in 2008 (Shao et al. 2008) and first named in 2011 (Li et al. 2011). Cycloxaprid can effectively control sucking and biting insects, especially imidacloprid-resistant populations of the brown planthopper (Shao et al. 2010). The mode of action of cycloxaprid was thought to be similar to that of imidacloprid, i.e., cycloxaprid was thought to affect the function of nicotinic acetylcholine receptors (Talley et al. 2008, Tomizawa et al. 2008, Ohno et al. 2009), but cycloxaprid’s exact mode of action remains unclear (Cui et al. 2012). Because it has performed well in controlling a broad spectrum of insect pests and has low toxicity for humans and livestock, cycloxaprid has been considered a substitute for imidacloprid for the control of agricultural insects in China (Shao et al. 2010). As a new insecticide, cycloxaprid requires additional study to clarify its mode of action and to determine whether it harms natural enemies, whether it develops resistance, and whether it exhibits cross-resistance with other insecticides.

Buprofezin is a thiadiazine insecticide that inhibits the synthesis of chitin (Izawa et al. 1985, Das et al. 2004), which is the main component of the insect cuticle. Buprofezin is mainly used to control homopteran pests (Gerling and Sinai 1994, Prabhaker and Toscano 2007, Yang and Yang 2007). Like cycloxaprid, buprofezin also has low toxicity against the human and leaves little residue in environment (Zhang et al. 2010). Although buprofezin kills *Nilaparvata lugens* nymphs during ecdysis (Izawa et al. 1985), its mode of action and the rate at which it kills target insects have not been well documented.

The white-backed planthopper (WBPH), *Sogatella furcifera*, is a devastating pest of rice worldwide (Cheng 2009, Sogawa et al. 2009). WBPH directly damages rice by feeding, which causes a characteristic yellowing of tissues known as “hopperburn,” and indirectly damages rice by vectoring a variety of plant pathogens such as southern rice black-streaked dwarf virus (Zhou et al. 2008). WBPH feeding can affect the content of amino acids in rice, the rate of photosynthesis, the level of chlorophyll, the activity of some enzymes, the growth rate, the time of tillering, and the filling of rice grain (Zhu and Cheng 2002, Suri and Singh 2011). In part, because it can reproduce and spread rapidly (Syobu et al. 2012), WBPH has become one of the most harmful planthoppers of rice (Atwal et al. 1967, Dhaliwal and Singh 1983). Control of WBPH has mainly relied on chemical insecticides (Endo and Tsurumachi 2001, Liu et al. 2002, Nizamani et al. 2002), and WBPH populations have developed resistances to many insecticides (Suri and Singh 2011).

Change of carboxylesterase (CarE) activity was regarded as an important mechanism for insecticide detoxification in insects (Oakeshott et al. 2005, Cao et al. 2008, Chang et al. 2010, Matsuda and Saito 2014). The covalent reaction of oxons with carboxylesterase 1, a serine hydrolase found in large quantities in the human liver, is one mechanism by which these compounds are detoxified and removed (Maxwell 1992). The glutathione S-transferases (GSTs) are a multifunctional family of phase II detoxification enzymes that protect cells against harmful endogenous toxic metabolites, superoxide radicals, and exogenous toxic chemicals (Buetler et al. 1995, Zimniak 2006, Allocati et al. 2009). To date, at least 14 classes of mammalian GSTs have been identified based on primary amino acid sequences (Hayes and Pulford 1995). Mixed function oxygenases (MFOs) metabolize a variety of xenobiotics such as polycylic aromatic hydrocarbons, drugs, and endogenous compounds such as steroids and fatty acids. Since the enzymes which constitute the MFO system decrease the lipid solubility of organic contaminants, thereby facilitating excretion (Jimenez and Stegeman 1990). The advantages of detoxification enzymes as biological indicators lie with their sensitivity as indicators of exposure.

Buprofezin is used extensively to control rice planthoppers in recent years in China due to the resistance increase of imidacloprid. Cycloxaprid is also studied to counteract the rise of resistance of rice planthoppers to other insecticides. However, action mechanism of these two insecticides is not clear at present. Therefore, we compared the toxicity (including the speed of action) of cycloxaprid and buprofezin against the WBPH and analyzed detoxifying mechanism in this research.

## Materials and Methods

### 

#### Rearing of WBPH

WBPH nymphs were supplied by Congfen Gao of Nanjing Agricultural University in 2010. They were reared on rice (cultivar Jinfeng provided by the Crop Institute, Shanghai Academy of Agricultural Sciences) in the laboratory at 26°C and with a photoperiod of 14:10 (L:D) h. The colony was not treated with any insecticides or any other pesticides.

#### Insecticides

Cycloxaprid (25%WP) was provided by the Shanghai Shengnong Pesticide Co. Ltd. Shanghai, China. Cycloxaprid is a new neonicotinoid insecticide that is produced by modifying the neonicotinoid insecticide IPP-10. It was discovered by the researchers of the East China University of Science and Technology (Shao et al. 2008, 2010). Buprofezin (37% SC) is a common insecticide and was produced by the Jiangsu Changlong Chemical Co. Ltd. Changzhou, China.

Cycloxaprid concentrations (indicated in terms of active ingredient) used in bioassays (see next section) ranged from 0 to 125 mg liter^−^^1^ for WBPH nymphs and adults. Buprofezin concentrations used in bioassays ranged from 0 to 148 mg liter^−^^1^ for WBPH nymphs and adults.

#### Bioassays

The toxicity of cycloxaprid and buprofezin to third-instar WBPH nymphs (easy treatment and count) and adult males and females was tested according to the method of Zhuang et al. (1999). Rice seedlings (cultivar Jinfeng) were grown in 29 by 19 by 9 cm plastic boxes containing sterilized water. When the rice seedlings were 6 cm tall, they were dipped in insecticide solution (three seedlings per insecticide concentration; solution concentrations are indicated in the Results) for 10 s, removed, and placed on filter paper to dry in the air. When dry, the three rice seedlings were placed in a 350-ml plastic cup containing plant growth nutrition solution (1 cm depth, NH_4_Cl: 155.10 mg liter^−^^1^, CaCl_2_: 110.75 mg liter^−^^1^, NaH_2_PO_4_•2H_2_O: 50.38 mg liter^−^^1^, K_2_SO_4_: 89.25 mg liter^−^^1^, MgSO_4_•7H_2_O: 405.00 mg liter^−^^1^, FeCl_3_•6H_2_O: 7.70 mg liter^−^^1^), and 30 nymphs or male or female adults, depending on the assay, were added to the cup by aspiration. The plastic cups were covered by black cloth that permitted gas exchange but prevented WBPH escape. Each treatment was represented by three replicate cups. The cups were maintained at the temperature and photoperiod as described in Rearing of WBPH section. The surviving nymphs and adults were counted every day for 1 wk. For the same concentration, the mortality on the 4th and 5th days was relatively high, so the toxicity of two insecticides to WBPH was tested on days 4 and 5 after treatment.

#### Specific Activity Tests of Three Detoxifying Enzymes

Ten surviving nymphal WBPHs on the 5th day at the higher concentrations of insecticides and control (no insecticide), respectively, were homogenized in 1,000 µl of ice-cold phosphate buffer (100 mM, pH 7.6) containing 0.1% Triton X-100. The homogenates were centrifuged at 4°C, 12,000 × *g* for 15 min, and the supernatants were collected to test the activity of three detoxifying enzymes. Protein concentrations were measured by the Bradford method (Bradford 1976) using bovine serum albumin as the standard.

##### CarE Assays

The CarE activity was measured referring to the method described by Van Asperen (1962) with some modifications and α-naphthyl acetate as a substrate. Hydrolysis reactions were performed at 37°C in a 96-well plate format in a total volume of 300 μl in 100 mM phosphate buffer (which had been adjusted to pH 7.6 at room temperature); 10 μl aliquot and 100 μl 100 mM phosphate buffer were removed and added to a 96-well plate format. α-Naphthyl acetate (0.3 mM) was prepared in ethanol. The reaction was started by adding 90 µl mixture solution containing 0.3 mM α-naphthyl acetate (containing eserine 0.3 mM). The mixture was incubated at 37°C for 30 min. Finally, 20 μl of 1% Fast Blue B salt:5% sodium dodeocyl sulphate (2:5 by volume) was added to each well using eight-channel multipipettes and incubated for 20 min at room temperature in the dark. The α-naphthol formation was measured at 600 nm using a spectrophotometer (Biotek Epoch). All samples were conducted in triplicate. One unit of activity was defined as the change of absorbance in per minute per milligram protein at 37°C.

##### GST Catalytic Activity Assays

The activity of GST toward the substrate 1-chloro-2,4-dinitrobenzene (CDNB) was determined according to the method of Habig et al. (1974). The conjugation was performed at 30°C in a 96-well plate format in a total volume of 300 μl in 100 mM phosphate buffer (which had been adjusted to pH 7.6 at room temperature). CDNB was prepared in ethanol. CDNB (0.6 mM) and 6 mM reduced glutathione were prepared in 190 μl 100 mM phosphate buffer. Aliquot (10 μl) was removed and added to the mixture to inspire the response. The mixture was incubated at 30°C for 5 min. The conjugation was measured at 340 nm using a spectrophotometer (Biotek Epoch). All samples were conducted in triplicate. One unit of activity was defined as the change of absorbance in per minute per milligram protein at 30°C.

##### MFO Assay

The MFO activity was measured referring to the method described by Li et al. (2006) with some modifications; 50 μl 1.0 mM p-Nitroanisole (pNA), 100 μl enzymatic aliquot, and 50 μl 1.0 mM NADPH were added to a 96-well plate format. The mixture was incubated at 37°C for 5 min. The formation was measured at 405 nm using a spectrophotometer (Biotek Epoch). All samples were conducted in triplicate. One unit of activity was defined as the change of absorbance in per minute per milligram protein at 37°C.

#### Statistical Analysis

LC_50_ values for the two insecticides and for three stages of WBPH (third-instar nymphs, adult females, and adult males) were calculated with probit analysis in SPSS 17.0. Specific activity of three detoxifying enzymes of surviving third-instar nymphs were compared by one-way analysis of variance in SPSS17.0. Means comparison method for the same detoxifying enzyme under different treatments is Tukey’s HSD (Honestly Significant Difference) test.

## Results

### 

#### The Effect of Cycloxaprid and Buprofezin on Survival of Nymphs and Adults of WBPH

**Fig. 1.**
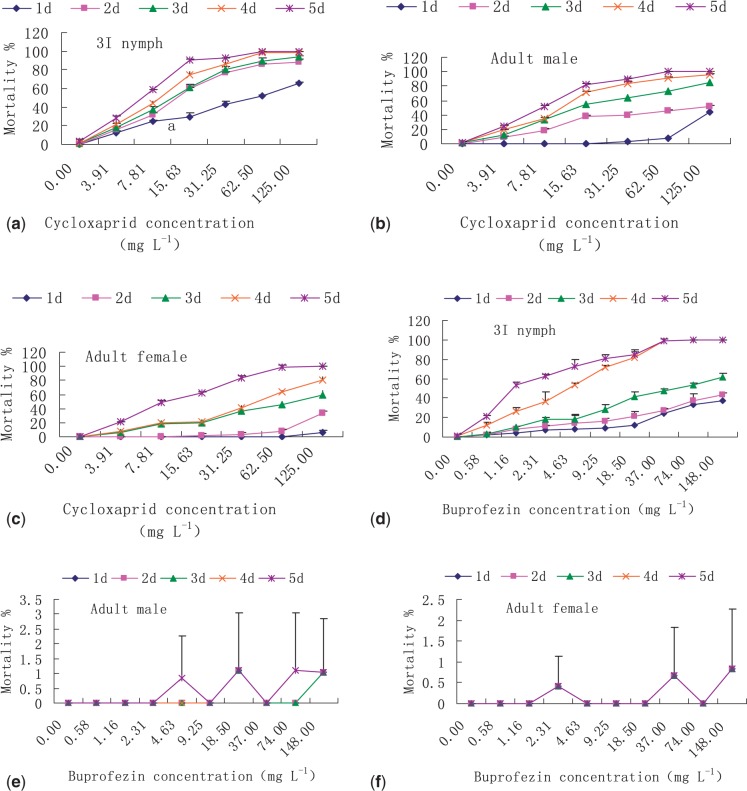
The effects of cycloxaprid and buprofezin on survival of WBPH nymphs and adults. (a) Cycloxaprid, third-instar (3I) WBPH nymphs. (b) Cycloxaprid, adult males. (c) Cycloxaprid, adult females. (d) Buprofezin, 3I WBPH nymphs. (e) Buprofezin, adult males. (f) Buprofezin, adult females. Values are means ± SD (*n* = 3).

Cycloxaprid caused high mortality of both WBPH nymphs and adults. With 125 mg liter^−^^1^ cycloxaprid, mortality of third-instar nymphs exceeded 65% after 1 day (Fig. 1a), adult male mortality exceeded 44% after 1 day (Fig. 1b), and adult female mortality exceeded 6% (Fig. 1c). In contrast to cycloxaprid, buprofezin caused only moderate mortality of nymphs and little or no mortality of adults 1 day after infestation. One day after infestation, mortality of third-instar nymphs was <38% with buprofezin at 148 mg liter^−^^1^ (Fig. 1d). A high percentage of adult WBPH remained alive even when exposed to the highest concentration of buprofezin (Fig. 1e and f).

Mortality of third-instar nymphs was generally higher after 2 d with cycloxaprid than with buprofezin (Fig. 1a and d). By the 4th day of exposure, however, mortality of third-instar nymphs was similar for the higher concentrations buprofezin and cycloxaprid (Fig. 1a and d). These results suggested that buprofezin was as effective but slower acting than cycloxaprid against third-instar WBPH nymphs. By the 4th day after infestation, cycloxaprid at 125 mg liter^−^^1^ caused ≥80% mortality of adults but buprofezin at 148 mg liter^−^^1^ (the highest rate tested) caused almost no adult mortality.

#### Toxicity of Cycloxaprid and Buprofezin for WBPH

For cycloxaprid, LC_50_ values on days 4 and 5 were lowest for third-instar nymphs, intermediate for adult males, and highest for adult females (Table 1). On day 5 and against WBPH third-instar nymphs, LC_50_ was about 3.79-fold lower for buprofezin than for cycloxaprid (Table 1).

**Table 1.** Survival of the nymphs (third instar, 3I) and adults of WBPH as a function of cycloxaprid and buprofezin concentration

Insecticide	WBPH stage^*a*^	Exposure time (d)	Regression equation^*b*^	χ^2^	df	*P*	LC_50_ (mg liter^−1^)	95% confidence limits
Cycloxaprid	3I nymph	4	*y* = −2.00+2.14*x*	0.57	3	0.90	8.68	0.18–20.33
		5	*y* = −2.10+2.64*x*	1.72	3	0.63	6.26	0.02–14.37
	Adult male	4	*y* = −1.92+1.87*x*	2.28	3	0.52	10.68	2.28–20.99
		5	*y* = −2.12+2.44*x*	1.82	3	0.61	7.42	0.28–15.51
	Adult female	4	*y* = −2.60+1.62*x*	0.45	3	0.93	40.19	0.21–74.81
		5	*y* = −2.59+2.48*x*	0.77	3	0.86	10.99	0.10–22.95
Buprofezin^*c*^	3I nymph	4	*y* = −1.57+2.17*x*	1.93	6	0.93	5.30	1.61–8.98
		5	*y* = −0.31+1.43*x*	3.86	6	0.70	1.65	0.02–5.97

Survival was determined 4 and 5 d after WBPHs were placed on treated rice leaves.

^*a*^3I refers to third instar.

^*b*^*y* refers to the probability value of WBPH mortality, and *x* refers to the common logarithm of pesticide concentration (mg liter^−1^).

^*c*^Regression equations for adults and buprofezin are not shown because buprofezin caused only low mortality of adults.

#### 3.3 The Activity of Three Detoxifying Enzymes

Mean CarE specific activity of nymphal WBPH treated with buprofezin was significantly higher than that of cycloxaprid and control, but there was no significant difference between cycloxaprid and control (*F* = 901.85, df = 2, *P* < 0.05). For GST and MFO, the comparison results were similar to CarE, and mean GST and MFO specific activity of nymphal WBPH treated with buprofezin was significantly higher than that of control and cycloxaprid (GST: *F* = 144.26, df = 2, *P* < 0.05; MFO: *F* = 8.37, df = 2, *P* < 0.05, see Table 2).

**Table 2.** Specific activity (mean ± SE) of three detoxifying enzymes of surviving 3I nymphal WBPH under 90% mortality pressure on the 5th day after treating with insecticides

Treatment	CarE (U min^−1 ^mg^−1 ^pro)	GST (U min^−1 ^mg^−1 ^pro)	MFO (U min^−1 ^mg^−1 ^pro)
Control	1.51 ± 0.04 b	0.94 ± 0.22 b	1.88 ± 0.30 b
Cycloxaprid	6.64 ± 0.08 b	1.19 ± 0.07 b	2.85 ± 1.24 b
Buprofezin	167.96 ± 5.45 a	41.47 ± 3.33 a	141.64 ± 48.08 a

Mean enzymatic activities followed by the same letter are not significantly different in the column (*P* > 0.05, Tukey’s HSD test).

## Discussion

In this study, both cycloxaprid and buprofezin caused substantial mortality of WBPH, although cycloxaprid was effective against both nymphs and adults, while buprofezin was effective only against nymphs. Our results are consistent with those of Zhuang et al. (2012), but unlike the study of Zhuang et al. (2012), our study focused on WBPH and provided more detail on cycloxaprid toxicity. In another study (Bo et al. 2008), the LC_50_ value for buprofezin against third-instar WBPH nymphs collected in Nanning, China, was 60 mg liter^−^^1^ on the 5th day of exposure, which was 36-fold greater than the LC_50_ value in this study. Our results for buprofezin were similar to those reported for WBPH collected in Jiangpu, Nanjing, China (Bo et al. 2008, Li et al. 2009). In those studies, buprofezin was reported to be an effective insecticides for control of the Nanjing population of WBPH. This study only measured the effect of cycloxaprid and buprofezin on WBPH mortality. Additional research is needed to determine the effects of cycloxaprid and buprofezin on WBPH oviposition, eclosion, and development, and different action mechanism.

Though the LC_50_ value for buprofezin was lower than that of cycloxaprid, the activity of three detoxifying enzymes treated with buprofezin was significantly higher than that of cycloxaprid. Therefore, we concluded that the better adaptability of WBPH to higher toxicity of buprofezin was due to the increase of activity of three detoxifying enzymes. Some individuals can survive longer under the pressure of insecticides. If these individuals cannot die, they will develop into insecticide-resistant populations and will be more difficult to control. The insecticide-resistant level of WBPH in field is positively correlated with the activity of the detoxifying enzymes, through which we can speculate the resistance degree of WBPH and make effective measurements to control them. However, after the cotton aphids were exposed to S,S,S-tributyl phosphorotrithioate, the CarE activity decreased gradually until 15 h and then gradually recovered until 24 h in the deltamethrin-selected-resistant strain (Chang et al. 2010). Cao et al. (2008) have reported the increased expression of the CarE due to the high transcription levels of CarE mRNA was related to deltamethrin resistance in cotton aphids. The conjugated 4-hydroxynonenal activity of rho-class GSTs from four aquatic species exhibited high activity toward this endogenous substrate (Leaver et al. 1993, Martinez-Lara et al. 2002, Doi et al. 2004, Carletti et al. 2008).

Overall, both cycloxaprid and buprofezin are effective insecticides for controlling WBPH. When rice fields are infested with many WBPH adults, we suggest application of cycloxaprid rather than buprofezin because cycloxaprid kills both nymphs and adults but buprofezin kills only nymphs. To avoid selecting for resistant populations of WBPH, we also recommend that the two insecticides should be applied alternately.
